# Diagnostic Accuracy of Post Procedural Creatine Kinase, MB Form can Predict Long-Term Outcomes in Patients Undergoing Selective Percutaneous Coronary Intervention?

**DOI:** 10.5812/cardiovascmed.11738

**Published:** 2014-02-24

**Authors:** Mohsen Maadani, Sepideh Parchami-Ghazaee, Ghodratollah Barati, Monireh Soltani, Elahe Amiri, Behshid Ghadrdoost, Mona Heidarali

**Affiliations:** 1 Cardiovascular Intervention Research Center, Rajaie Cardiovascular Medical and Research Center, Iran University of Medical Sciences, Tehran, IR Iran; 2 Cardiac Electrophysiology Research Center, Tehran University of Medical Sciences, Tehran, IR Iran; 3 Rajaie Cardiovascular Medical and Research Center, Iran University of Medical Sciences, Tehran, IR Iran

**Keywords:** Creatine Kinase, MB Form, Angioplasty, Balloon, Coronary, Myocardial Infarction

## Abstract

**Background:**

Measuring cardiac markers in blood has been the main strategy for the diagnosis of acute myocardial infarction for nearly 50 years. Creatine kinase-MB (CK-MB) has been demonstrated to be a highly specific marker.

**Objectives:**

The present study aimed to assess the role of CK-MB changes following percutaneous coronary intervention (PCI) to predict one year outcomes of this procedure.

**Patients and Methods:**

This cohort study was conducted on 138 patients diagnosed with coronary artery disease who underwent PCI. Sixty-nine patients who had a CK-MB elevation ≥ 3 times upper limit of normal (ULN) post procedurally were considered as group I and 69 patients without cardiac enzyme rise after PCI were considered as the control group (group II). The composite end point of major adverse cardiac events (MACE) during one year was assessed by telephone follow-up or presentation at clinical visiting, and compared between the two groups. The MACE was defined as the appearance of at least one of the following events: mortality, repeated revascularization procedures, myocardial infarction, or cerebrovascular events.

**Results:**

Although one year mortality in the group I was 4 (5.8%), about two times greater than the other group 2 (2.9%), the difference was not significantly discrepant (P = 0.57). Moreover, 8 (11.6%) of patients in group I experienced one year MACE, while this rate in the other group was 4 (5.8%), with insignificant difference (P = 0.22). In group I, one case experienced coronary artery bypass surgery, one, exhibited cerebrovascular disease and one reported ST segment elevation myocardial infarction (STEMI), while two patients in the other group were suspicious of having non-ST segment elevation myocardial infarction (NSTEMI) and candidates for repeated PCI. Multivariate analysis revealed that increased post-procedural CK-MB ≥ 3 times UNL could not predict long-term MACE in patients who underwent selective PCI. Area under the curve (AUC) for predicting one year MACE was 0.593 (95% CI: 0.397 - 0.788), indicating inappropriate accuracy for this biomarker (P = 0.290).

**Conclusions:**

It seems that CK-MB ≥ 3 times ULN within 24 hours after PCI cannot independently predict one year MACE in patients undergoing PCI.

## 1. Background

Biochemical markers have been useful non-invasive diagnostic tools in the diagnosis of both ischemic and non-ischemic myocardial injury. Although ischemic events can be easily identified on 12-lead electrocardiograms (ECG), these specific biomarkers can increase diagnostic accuracy. Measuring cardiac markers in blood has been the main strategy for the diagnosis of acute myocardial infarction (MI) for nearly 50 years. It has been demonstrated that creatine kinase-MB (CK-MB) is a highly specific marker (1, 2). Myocardial injury may occur in association with procedures on the coronary circulation, including percutaneous coronary intervention (PCI) or coronary artery bypass graft surgery. Since biomarker release in the setting of PCI is a reflection of increased baseline risk in the patients, in which it occurs, considering and assessing the trend of the changes of these biomarkers may be valuable to predict adverse outcomes (3). Although after elective PCI, elevations of cardiac troponins occur more frequently than elevations of CK-MB, one of the independent variables which seem to be associated with an increased long-term mortality rate is CK elevation > 750 mg/dL (4). However, the role of CK-MB elevation in survival after non-surgical coronary interventions is controversial.

## 2. Objectives

The present study aimed to assess the role of CK-MB changes following PCI to predict one year outcomes of this procedure.

## 3. Patients and Methods

This descriptive cohort study was conducted on 138 patients with a diagnosis of coronary artery disease who underwent PCI between February 2011 and December 2012 at Tertiary Center of Iran University of Medical Sciences, Tehran, Iran. The inclusion criteria were normal pre-procedural cardiac troponin I (cTnI) and CK-MB levels. Patients with a diagnosis of recent MI were excluded. The diagnosis of a pre-procedural MI was based on either the development of new pathological Q waves in at least two continuous ECG leads or an elevation of CK-MB > five times the upper limit of normal (ULN) or cTnI > 0.06 ng/mL. Sixty nine patients had CK-MB elevation ≥ 3 times ULN post procedurally (5), which were considered as group I (n = 69) and 69 patients (group II) without cardiac enzyme rising after PCI, considered as a control group. The composite end point of major adverse cardiac events (MACE) during one year was assessed by telephone follow-up or presentation at clinical visiting, and compared between groups. The MACE was defined as the appearance of at least one of the following events: mortality, repeated revascularization procedures, myocardial infarction or cerebrovascular events. The standard PCI technology was utilized. All patients provided written, informed consent to study participation and the study protocol was approved by the Institutional Review Board and the Ethics Committee of Iran University of Medical Sciences, Tehran, Iran. For the statistical analysis, IBM SPSS Statistics® 19.0 for Windows® (IBM Corp, Armonk, NY, USA) was used. Results were reported as mean ± standard deviation (SD for the quantitative variables and count (percentage) for the categorical ones. Two groups were compared using the Student's t-test or Mann-Whitney U test for the continuous variables and the chi-square test (or Fisher's exact test, if required) for the categorical variables. The ROC curve was used to identify the role of CK-MB for discriminating MACE. A P value < 0.05 was considered statistically significant.

## 4. Results

Clinical characteristics of the study population and procedural findings are shown in Table 1. The two groups were matched in terms of gender, as well as general cardiac risk factors. However, the patients in group I were slightly older. There were no considerable changes regarding stent characteristics regarding the number and type of the applied stents between the two groups. Although 1-year mortality in group I was 4 (5.8%) - about two times greater than the other group 2 (2.9%), the difference was not significantly discrepant (P = 0.57).

**Table 1.** Baseline Clinical and Procedural Characteristics of the Patients According to Post PCI CK-MB Status ^a^

Characteristics	CK-MB ^b^ ≥ 3 times, n = 69	CK-MB ^b^ < 3 times, n = 69	P value
**Male**	47 (68.1)	46 (66.7)	0.856
**Age, y**	60.72 ± 10.31	57.19 ± 10.46	0.048
**Smoking cigarette**s	13 (18.8)	20 (29.0)	0.162
**Diabetes mellitus**	22 (32.4)	15 (21.7)	0.162
**Family history of CAD ** ^**a**^	9 (13.0)	14 (20.3)	0.253
**Hypertension**	26 (37.7)	34 (49.3)	0.170
**Hyperlipidemia**	31 (44.9)	37 (53.6)	0.170
**Stent number**			0.987
1	44 (63.8)	44 (63.8)	
2	20(29.0)	19 (27.5)	
3	4 (5.8)	5 (7.2)	
4	1 (1.4)	1 (1.4)	
**Type of stent**			0.492
Bare-metal stents	41 (59.4)	28 (40.6)	
Drug-eluting stents	37 (53.6)	32 (46.4)	

^a^ Data are presented as No (%) or mean ± SD.

^b^ Abbreviations: CAD, coronary artery disease; CK-MB, creatine kinase-MB.

**Figure 1.**
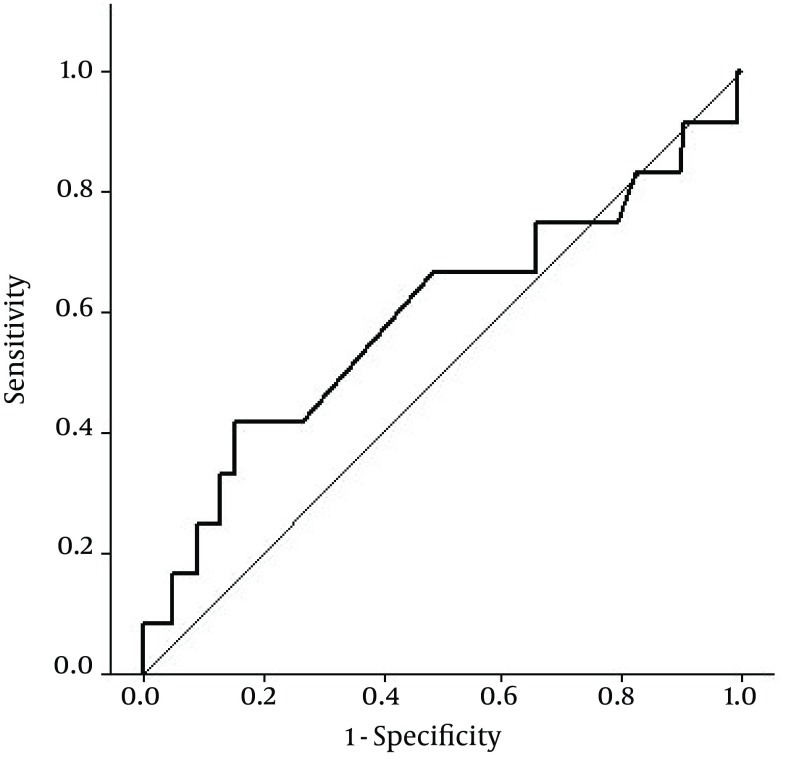
Receiver Operative Characteristics (ROC) Curves for Determining the Accuracy of CK-MB Enzyme to Discriminate Major Adverse Cardiac Events

In addition, 8 (11.6%) of patients in the group I experienced one year MACE, while this rate in group II was 5.8%, with insignificant difference (P = 0.227). In the group with post procedural CK-MB ≥ 3 times ULN, one case experienced coronary artery bypass surgery, one exhibited cerebrovascular disease and one was reported with STEMI. However, two patients in the other group were suspicious of having non-ST segment elevation myocardial infarction (NSTEMI) and candidates for repeating PCI. Multivariable analysis after adjustment for different independent variables, which are mentioned in Table 1, showed that increased post-procedural CK-MB ≥ 3 times UNL could not predict long-term MACE in patients who underwent selective PCI. The accuracy of post procedural CK-MB in predicting one year MACE was examined using ROC curve analyses (Figure 1). Area under the curve (AUC) for predicting one year MACE was 0.593 (95% CI: 0.397 - 0.788), indicating inappropriate accuracy for this biomarker (P = 0.290).

## 5. Discussion

The goal of the present study was to assess the ability of CK-MB biomarker in predicting long term outcome following selective PCI. This observation, that there is an association between elevated biomarkers after PCI and worsened long term outcomes, was first made regarding CK-MB (6). It has been reported that CK or CK-MB elevation occurs in 25% of patients undergoing PCI (7). We considered an increased level of this biomarker higher than three times of normal range within 24 hours after procedure as the target criterion. Although this is the most common definition of periprocedural MI, it obviously seems to be an arbitrary cutoff. In our study, we could not finally demonstrate the efficacy of CK-MB for predicting long-term MACE in affected patients. According to Cutlip and Kuntz (7), a variety of cutoff points for the definition of increased level of CK-MB may be considered. Although even a CK elevation of 1.5 to 3 times the ULN is associated with increased mortality, each 100 U/L increase of CK is associated with 1.05 times enhanced relative risk of cardiac mortality. It is important to note that some authors even considered CK elevations more than 5 or 8 times the ULN to find any association with poor adverse outcomes. Furthermore, other indicators, like the time of biomarker measurement (before and within, as well as early and late after PCI) and follow-up period can also be effective in the efficacy of cardiac-specific markers for predicting adverse outcomes following PCI (7).

In this regard, our study revealed that the determination of the post PCI CK-MB changes within 24 hours after the procedure could not predict one year MACE. The results of some previous studies agree with our observation, whereas others are inconsistent with our survey. Chia et al. (8) have demonstrated that in a study on 378 patients undergoing PCI, among three markers of CK, CK-MB, and troponin I, only the last one could potentially predict mid-term MACE in these patients. Byrne et al. (9) showed that both CK-MB and troponin I could indicate long term adverse events, yet the predictive value of troponin was considerably higher than what was reported. Meanwhile, in their study, only two parameters of MACE; mortality and occurrence of myocardial infarction, were considered as study endpoints. In another research by Roe et al. (10), which only included NSTEMI patients, the peak range of CK-MB was determined as a main predictor of six-month mortality. Although in univariate analysis, raised CK-MB could be associated with long-term MACE like in the present study, considering increased CK-MB more than 3 times of ULN within 24 hours after PCI, in a multivariable model however, only stent size and diameter could markedly predict long-term MACE (11).
